# Muscle-Specific Sensitivity to Voluntary Physical Activity and Detraining

**DOI:** 10.3389/fphys.2019.01328

**Published:** 2019-10-23

**Authors:** Jon-Philippe K. Hyatt, Emily A. Brown, Hannah M. Deacon, Gary E. McCall

**Affiliations:** ^1^College of Integrative Sciences and Arts, Arizona State University, Tempe, AZ, United States; ^2^Department of Exercise Science, University of Puget Sound, Tacoma, WA, United States

**Keywords:** soleus, plantaris, myosin heavy chain, capillary, citrate synthase, OXPHOS, rat

## Abstract

Aerobic physical activity triggers adaptations in skeletal muscle including a fast-to-slow shift in myosin heavy chain (MHC) isoforms, an enhanced capillary network, and mitochondrial biogenesis to meet increased demands placed upon this tissue. Although the magnitude of these responses appears to be dependent on muscle phenotype as well as training volume and/or intensity, the whole-muscle response to detraining remains mostly unexplored. Here, we hypothesized that the shifts toward slower MHC phentotype and the increased capillarity and mitochondrial oxidative markers induced with training would return toward sedentary (SED) control levels sooner in the fast plantaris than in the slow soleus muscle as a result of detraining. Soleus and plantaris muscles from 8-week (TR 8wk) voluntarily running adult female Sprague–Dawley rats were compared to muscles from SED and detrained rats (DETR) (4 weeks voluntary running followed by 4 weeks of reduced activity), which were subdivided into low- (DETR Lo) and high-running-distance (DETR Hi) groups. We show that maintaining the fast-to-slow MHC isoform shift required consistent aerobic training in the soleus and plantaris muscles: detraining clearly abolished any fast-to-slow gains in the plantaris, whereas the training volume in DETR Hi rats appeared to influence the MHC return to basal levels in the soleus. Total capillary number (per mm^2^) in the plantaris increased in all groups compared to SED levels, but, in the soleus, this enhancement was observed only in the TR 8wk rats. Generally, increased mitochondrial markers for aerobicitiy were observed in TR 8wk plantaris, but not soleus, muscles. In a second experiment, we show that the muscle-specific adaptations were similar after 4 weeks of voluntary exercise (TR 4wk) as in 4 weeks (TR 8wk). Taken together, our findings suggest that the plantaris muscle is more sensitive to voluntary physical activity and detraining than the soleus muscle; these results also demonstrate that the soleus muscle requires a greater aerobic challenge (i.e., intensity, duration) to trigger phenotypic, angiogenic, or aerobic enzyme adaptations. Our findings generally suggest that muscular aerobic fitness to voluntary running, or its loss during detraining, manifests as changes occurring primarily within fast, rather than slow, muscle phenotypes.

## Introduction

Skeletal muscle adaptation to aerobic-endurance training is, in part, characterized by enhancements in oxygen consumption, blood flow, and a propensity for myosin heavy chain (MHC) isoforms to shift from fast-glycolytic to slow-oxidative phenotypes (Holloszy, 1967; Armstrong and Laughlin, 1984; Coyle et al., 1984; Ishihara et al., 1991; Montero et al., 2015). In rodent models of endurance training, skeletal muscles generally increase in slow (type I) and/or more oxidative (types I and IIa) and decrease in fast/glycolytic (types IIx and IIb) MHC isoforms (Fuller et al., 2006). Fast-to-slow MHC phenotypic shifts with exercise appear to be dose-dependent (Demirel et al., 1999; Allen et al., 2001; Seene et al., 2005), which may explain differences observed between forced versus voluntary endurance training in animal models since these approaches differ in running intensity and duration (Bigard et al., 2000; Serrano et al., 2000; Allen et al., 2001; Kadi et al., 2002; Waters et al., 2004; Seene et al., 2005; Jeneson et al., 2007; Glaser et al., 2010). The impact of exercise on MHC isoform expression has been reviewed extensively elsewhere (Baldwin and Haddad, 2001; Booth et al., 2015).

The rodent soleus muscle is characteristically fatigue-resistant and exhibits a greater capacity for oxidative metabolism, which is attributed to a high proportion of type I MHC and greater capillary and mitochondrial densities compared to other hindlimb muscles. These contractile and metabolic properties support the high levels of soleus recruitment in rats; the soleus is, by far, the most active hindlimb muscle during routine daily cage activity compared to other plantarflexors (Alford et al., 1987; Hodgson et al., 2005). The majority of this daily activation appears to be used for postural positioning rather than locomotion. The forces required for locomotion, as well as to change velocities for walking, running or sprinting are generated from phenotypically faster hindimb muscles, including the tibialis anterior, medial gastrocnemius, and plantaris muscles (Gardiner et al., 1986; Hodgson et al., 2005).

Muscle perfusion at rest also is disproportionate between the soleus and plantaris and coincides with their aforementioned recruitment patterns and metabolic activity; the untrained soleus receives ∼4.5-fold greater blood flow than the plantaris under non-exercising conditions (Laughlin and Armstrong, 1982). Acute treadmill running enhances blood flow to both muscles: although the soleus receives 26–62% more blood than at resting levels, blood flow to the exercising plantaris is enhanced by 300–750% (Laughlin and Armstrong, 1982). Long-term aerobic exercise training increases capillary density in both fast and slow muscles (Klausen et al., 1981; Coyle et al., 1984; Malek et al., 2010; Olenich et al., 2013, 2014), which is reflected by changes to resting perfusion of fast and slow trained muscles. Soleus resting blood flow increases ∼18% after 13–17 weeks of graded forced treadmill training, whereas blood flow in the plantaris increases ∼250% (Armstrong and Laughlin, 1984), suggesting that the relative angiogenic response to aerobic exercise is muscle-specific.

Finally, endurance training disproportionately affects the mitochondrial content of fast and slow muscles (Fuller et al., 2006; Hamidie et al., 2015; Yokokawa et al., 2018). Based on the inherent differences in muscle recruitment and perfusion at rest or during low level activity, the untrained soleus is supported by a greater oxidative metabolic capacity compared to the plantaris. The exercise-trained plantaris experiences a larger change in mitochondrial markers and aerobic enzymatic activity compared to the soleus (Yokokawa et al., 2018). Taken together, the magnitude of change in fast-to-slow phenotypic MHC shifts, capillarity, and oxidative characteristics to endurance exercise training is greater in the plantaris than in the soleus because of the disparities that exist between these muscles during untrained, sedentary (SED) conditions. In effect, the plantaris has a greater potential to enhance its aerobic capacity, and exhibits a greater acute physiological shift in perfusion during exercise than the soleus.

Detraining is a reduction or cessation in exercise frequency, intensity, or duration required to maintain the physiological gains achieved during exercise training (Mujika and Padilla, 2000, 2001). In general, the time course of losing training adaptations is dependent on the initial level of fitness, the physiological system, and the length of detraining. Although limited individual studies have examined the impact of detraining on overall aerobic fitness (Coyle et al., 1984) or capillarity (Klausen et al., 1981; Madsen et al., 1993; Malek et al., 2010; Olenich et al., 2014; Prior et al., 2015), the muscle-specific changes and temporal response to detraining are unexplored. Given that endurance exercise triggers a more robust acute and chronic response in the relatively fast plantaris than the slow soleus muscle, we hypothesized that adaptations in MHC phenotypes, the microvasculature, and mitochondrial markers would return to SED levels in the plantaris and not in the soleus as a result of detraining and irrespective of the total voluntary running distance accrued. We also expected that the regression of these adaptations in the detrained plantaris would return to control (e.g., SED) levels based on the presumption that adaptations garnered during training are more immediately expendable in the detrained plantaris given its lower level of recruitment during SED conditions (Hodgson et al., 2005). Conversely, we anticipated that any training adaptations achieved by the soleus would be sustained throughout a detraining period because of its sustained high level of recruitment among the hindlimb muscles.

## Materials and Methods

### Animals and Physical Activity Training Protocols

**FIGURE 1:**
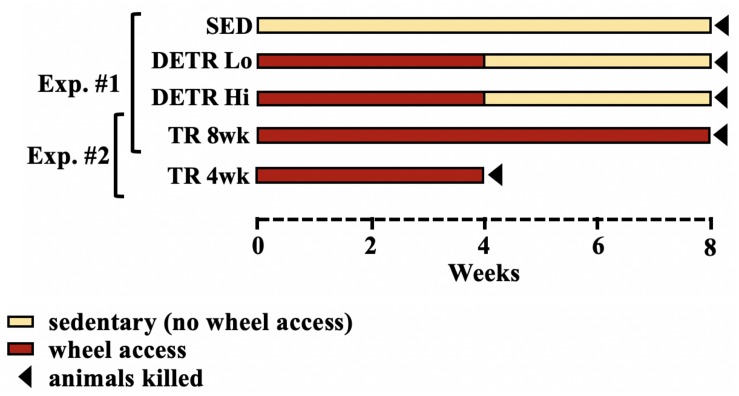
Schematic representation of experimental design. In the first experiment (Exp. #1), sedentary (SED), detrained (DETR), and exercised-trained (TR 8wk) rats were compared over an 8-week period. DETR rats accessed resistance-free running wheels for 4 weeks before returning to SED conditions for an additional 4 weeks and were divided into low (DETR Lo) and high (DETR Hi) running distance groups. In the second experiment (Exp. #2), rats accessed wheels for 4 weeks (TR 4wk) and were sacrificed at the end of this period and independently compared to TR 8wk rats based on similar total running distances. Sedentary conditions for SED and DETR groups refer to normal cage activity associated with standard rodent housing (see section “Materials and Methods”).

All procedures and treatment protocols were approved by the University of Puget Sound Institutional Animal Care and Use Committee in accordance with the guidelines of the American Physiological Society. Early-adult Sprague–Dawley female rats (Harlan Labs, Frederick, MD, United States) (∼120 g) were given 48 h to acclimate to new housing upon receipt and *ad libitum* access to standard rat chow and water. This study was conducted in two parts (Figure 1).

#### Experiment #1

Rats were randomly assigned to three groups (*n* = 8–10/group). A SED control group was housed as pairs in standard cages for 8 weeks. An exercise-trained group (TR 8wk) consisted of rats that were housed individually in cages with voluntary access to resistance-free running wheels (Lafayette Instrument, Lafayette, IN, United States) 8 weeks. Daily running distances were recorded by Activity Wheel Monitor v11.12 software (Lafayette). Finally, detrained rats (DETR) had access to running wheels for 4 weeks followed by a 4-week detraining period in which the rats were housed in standard cages identical to the SED rats. Using an independent *t*-test, a significant difference in DETR running distances was detected between low- and high-distance runners within this group (*p* < 0.05) which were subsequently segregated into DETR Lo and DETR Hi groups, respectively (Figure 1). It should be noted that, for a separate study investigating global gene expression (data not shown), the SED, DETR Lo, and TR 8wk rats underwent a 1-h moderate (20 m/min) forced running challenge 24 h prior to termination. This acute running challenge was assumed to not have an impact on the profiles of proteins examined in the present study, which reflect the net accumulation in protein following an 8-week experimental protocol.

#### Experiment #2

In an attempt to observe the muscle-specific adaptations in DETR rats during their exercise-training period, a 4-week-trained group (TR 4wk) was initially included for comparison. Unexpectedly, exercising TR 4wk rats ran considerably greater distances than either the DETR Lo or DETR Hi groups, which made group comparisons untenable. Because TR 4wk rats ran a similar total distance in 4 weeks as the TR 8wk rats ran in 8 weeks, our muscle-specific observations made in TR 4wk rats were only compared with the TR 8wk rats only (Figure 1).

At the end of the experimental period for each group (Figure 1), rats were deeply anesthetized with a lethal overdose of pentobarbital sodium (Euthasol) and the soleus and plantaris muscles from each rat were removed bilaterally, trimmed of excess connective tissue, wet weighed, pinned to cork at the approximate *in situ* resting length, and frozen in isopentane cooled by liquid nitrogen. All tissue samples were stored at −80°C until further analysis.

### Myosin Heavy Chain Isoform Separation

Skeletal muscle portions near the mid-belly (25–50 mg) were bead homogenized for 2.5 min at 3,000 rpm in 10-volume ice-cold buffer (pH 6.8) containing 10 mM Tris–HCl, 5 mM EDTA, 0.25 M sucrose, 100 mM KCl, 0.5% Triton-X, and 1 mM dithiothreitol, 3 mM benzamidine, 1 mM sodium orthovanadate, 10 mM leupeptin, 5 mg/ml aprotinin, and 1 mM 4-[(2-aminoethyl) benzenesulfonyl fluoride]. After homogenization, the samples were separated into two aliquots for MHC or total protein isolations. The MHC aliquot was centrifuged at 1,000 × *g* for 10 min at 4°C and the pellet resuspended in ice-cold wash buffer (10 mM Tris–HCl, 2 mM EDTA, 175 mM KCl, 0.5% Triton-X; pH = 6.8) and centrifuged again at 1,000 × *g* for 5 min at 4°C. Finally, the pellet was washed in ice-cold buffer (10 mM Tris–HCl and 150 mM KCl; pH = 7.0), centrifuged at 1,000 × *g* for 5 min at 4°C and the pellet was resuspended in 50 μl of the same buffer. The total protein aliquot was transferred to clean tubes immediately following homogenization and centrifuged at 12,000 × *g* for 10 min at 4°C. The supernatant was transferred to clean tubes in 50 μl aliquots and frozen at −80°C for enzyme assays and Western analyses. MHC and total protein concentrations were determined using the Bio-Rad Protein Assay (Bio-Rad, Hercules, CA, United States) as per the manufacturer’s instructions.

Myosin heavy chain isoforms in soleus and plantaris muscles were separated using standard methods outlined by Talmadge and Roy (1993). Briefly, MHC isolates from each sample were boiled for 2 min at 100°C in sample buffer and 12 μg of each sample was loaded into wells of a 1.5-mm-thick 8% SDS–PAGE gel (using a 50:1 acrylamide:bis-acrylamide ratio) that was then subjected to 60 V for 1 h followed by 85 V for 22–24 h at 4°C. A standard marker prepared from rat medial gastrocnemius muscles was loaded in a separate lane to ensure that all four adult MHC isoforms could be identified in each gel. Following electrophoresis, the proteins were fixed in 12.5% trichloroacetic acid for 10 min, rinsed in ddH_2_O, and the gels were stained with quick Coomassie Blue R250 (Sigma) for 1 h and de-stained with ddH_2_O. The identification of each MHC isoform band within the gels was in accordance with well-established reports (Haddad et al., 1993; Talmadge and Roy, 1993; Demirel et al., 1999; Kariya et al., 2004; Fuller et al., 2006; Hyatt et al., 2010). Each gel was scanned and the separated MHC bands were quantified using ImageJ (Rasband et al. (1997–2018)). The amount of each MHC isoform is expressed as a percent of the total MHC protein detected in each sample. Finally, gels were mounted on a drying frame for 24 h and stored.

### Capillary Density

The soleus and plantaris muscles contralateral to the side used for protein analysis were used for histology. Four to six 10 μm-thick serial cross-sections were obtained from each frozen muscle near the mid-belly using a cryostat (Leica CM1950) at -18°C and mounted on gelatin-coated slides. To visualize capillaries, a lead-based ATPase staining procedure was used according to earlier work (Rosenblatt et al., 1987). Muscle sections were fixed for 5 min at 4°C in a 5% formalin (v/v) buffered solution (pH 7.6) containing 0.144 M sodium cacodylate, 0.068 M CaCl_2_, and 0.336 M sucrose. Samples were rinsed with ddH_2_O and then incubated at 37°C for 60 min in a freshly prepared medium containing 6.25% gelatin (w/v), 0.048 M Tris–HCl, 0.661 M ATP, 0.00375 M lead nitrate, and 0.00708 M calcium chloride. The samples were gently swirled in the lead-based incubation medium every 15 min. Following a rinse in ddH_2_O, samples were developed in 2% ammonium sulfide for 1 min and then wet-mounted in Kaiser Glycerin Jelly, containing 50% glycerol and 8% gelatin, before analysis. Capillaries appeared as brown-black structures adjacent to fibers.

Stained cross-sections for all samples were viewed using bright field microscopy (EVOS FL Auto 2 Cell Imaging System; Thermo Fisher Scientific, Waltham, MA, United States) and images were captured under the 20X objective lens. Two fields per sample were captured to include a total of 100–150 contiguous muscle fibers that were free of large connective tissue spaces. Only whole or near-whole fibers within the captured field of view were counted. All group identifiers were masked to avoid biases from three independent counters who assessed the number of capillaries and fibers per field. Occasionally, capillaries appeared in an oblique orientation; in such instances, those capillaries were counted as a single vessel. For each sample, the total capillary number was normalized to a square millimeter, averaged per group, and compared.

### Citrate Synthase Enzyme Activity

All frozen samples were thawed at 4°C and all solutions used in this assay (Srere, 1969) were kept at 30°C. The spectrophotometer was set at 412 nm wavelength and programmed to maintain a constant temperature at 30°C. Pre-warmed 0.325 ml 100 mM Tris buffer (pH 8.0), 50 μl DTNB (55′-dithiobis-(2-nitrobenzoic acid), Sigma, St. Louis, MO, United States), 50 μl Acetyl CoA (Sigma) were added to each cuvette. Soleus and plantaris muscle samples were diluted 1:100 and 1:50, respectively, in 100 mM Tris buffer (pH 8.0) just before use. Dilute sample homogenate was added (25 μl) to the cuvette and the reactions were initialized with the addition of 50 μl of oxaloacetic acid (Sigma) and mixed by gentle pipetting. The OD_412_ was recorded every minute for 5 min and the average change in OD for the last 4 min was used to calculate the citrate synthase activity. Activity (μmole/g/min) was calculated as: [(Δ OD/minute × dilution factor × 20 × 20)/13.6] and then normalized to protein concentration of each sample (nmol/mg protein/min) and used for comparisons between groups.

### Western Blot Analyses

Loading for immunoblotting was determined to be 15, 10, and 5 μg/sample for citrate synthase, oxidative phosphorylation (OXPHOS) and glyceraldehyde 3-phosphate dehydrogenase (GAPDH; loading control) proteins, respectively. A biotin-conjugated molecular weight marker (Cell Signaling, Beverly, MA, United States) was used to confirm the target proteins by size. Samples were loaded according to muscle type and included a representation of all treatment groups for each gel. Protein was denatured by heating samples in SDS–PAGE sample buffer (0.2% SDS, 20% glycerol, 25% 4X buffer, 5% β-mercaptoethanol, and 0.025% bromophenol blue) at 42–44°C for 3 min and electrophoresed in an SDS-12% polyacrylamide gel at 85 V for 20–25 min and then 135 V for 80 min. The proteins were transferred to PVDF membranes for 3 h at 60 V (∼425 mA total). Next, the membranes were placed in a solution of Ponceau S (Sigma) to verify that protein loading was similar between samples and that the transfer was uniform and artifact-free (data not shown) before immersed in a blocking solution containing 5% nonfat dry milk (NFM) dissolved in phosphate buffered saline with 0.05% Tween-20 (T-PBS) for a minimum of 0.5 h. All antibodies were diluted in blocking solution and the membranes were incubated in either rabbit anti-GAPDH (1:30,000; Proteintech, Rosemont, IL, United States), rabbit anti-citrate synthase (1:2,000; ab96600, Abcam, Cambridge, MA, United States), or mouse anti-OXPHOS (1:40,000; Abcam) antibodies for 1 hr at room temperature. The primary OXPHOS antibody (ab110413) is a five-in-one mouse monoclonal antibody cocktail containing: a subunit of mitochondrial NADH (NDUFB8, ab110242); the iron-sulfur subunit of mitochondrial succinate dehydrogenase (SDHB, ab14714); mitochondrial cytochrome b-c1 complex subunit 2 (UQCRC2, ab14745); mitochondrial-encoded cytochrome c oxidase I (MTCO1, ab14705); and the alpha subunit of mitochondrial ATP synthase (ATP5A, ab14748). The membranes were washed for 1 h in several changes of T-PBS and then incubated for 1 h at room temperature in horseradish peroxidase-linked secondary antibodies including, anti-mouse IgG (1:10,000) for OXPHOS targets, anti-rabbit IgG for citrate synthase (1:15,000) and 1:20,000 for GAPDH targets, and anti-biotin (1:1,000) the molecular weight marker. Finally, the membranes were washed for 1 h in T-PBS and then developed using an ECL detection kit (Amersham, Piscataway, NJ, United States) per the manufacturer’s instructions. Exposure to Kodak X-Omat film was either ∼5, 10–12, or 35–40 s for GAPDH, citrate synthase, or OXPHOS targets, respectively. For each protein, densitometry and quantification were performed using ImageJ. The adjusted relative density was ascertained using GAPDH as the loading control for each OXPHOS protein, averaged, and compared between groups.

### Statistics

Values are presented as means ± SE. For Experiment #1, a one-way analysis of variance (ANOVA) was performed to ascertain overall differences for SED, DETR Lo, DETR Hi, and TR 8wk groups for all variables and a Tukey’s one-tailed test was used for *post hoc* comparisons. For Experiment #2, an independent two-tailed *t*-test (equal variances assumed) was employed to detect any differences between TR 4wk and TR 8wk rats. For all statistical analyses, SPSS (v24) was used and significance was set at *p* < 0.05.

## Results

### Experiment #1

#### Physical Activity and Muscle Mass

Total voluntary running distances and mean body and muscle masses for each group in Experiment #1 are shown in Table 1. DETR Lo rats ran significantly less total distance in 4 weeks than DETR Hi rats and TR 8wk rats at the 4-week time point (*p* < 0.05). However, after 4 weeks of running DETR Hi and TR 8wk distances were similar. At the end of the experiment, the mean body mass of the TR 8wk rats was less than SED, DETR Lo, and DETR Hi rats (*p* < 0.05), respectively. The mean soleus absolute muscle mass was lower in DETR Lo than DETR Hi (*p* < 0.05) and the mean soleus mass relative to body mass was lower in DETR Lo rats than SED, DETR Hi, and TR 8wk rats (*p* < 0.05). Mean plantaris absolute mass was lower in TR 8wk rats than SED, DETR Lo, and DETR Hi rats (*p* < 0.05), whereas plantaris masses relative to body mass were similar among groups.

**TABLE 1:** Mean (±SE) total running distance, ending body mass, and soleus and plantaris absolute and relative muscle masses in adult female Sprague–Dawley rats after either sedentary (SED), detrained (DETR), or trained (TR) conditions.

			**Soleus mass**		**Plantaris mass**	
				
**Group (n)**	**Total running**	**Body mass**	**Absolute**	**Relative**	**Absolute**	**Relative**
	**(km)**	**(g)**	**(mg)**	**(mg/g)**	**(mg)**	**(mg/g)**
SED (8)	0 ± 0	247 ± 9	118.3 ± 5	0.48 ± 0.01	305.1 ± 12.2	1.23 ± 0.02
DETR Lo (8)	145.9 ± 14.1	247 ± 5	107.6 ± 2.9 a	0.44 ± 0.01 a	299.9 ± 6.6	1.22 ± 0.02
DETR Hi (9)	312.4 ± 40.2 b	244 ± 3	115.7 ± 4.1 b	0.47 ± 0.01 b	297.3 ± 11.6	1.22 ± 0.04
TR 8wk (8)	560.3 ± 71.2 b,c	225 ± 8 a,b,c	109.8 ± 6.5	0.49 ± 0.01 b	276.8 ± 12.3 a,b,c	1.23 ± 0.02
	265.2 ± 41.2 b^∗^					

*^∗^Mean running distance for TR 8wk rats after 4 weeks. a – different vs. SED (*p* < 0.05); b – different vs. DETR Lo (*p* < 0.05); c – different vs. DETR Hi (*p* < 0.05).*

#### MHC Isoforms

**FIGURE 2:**
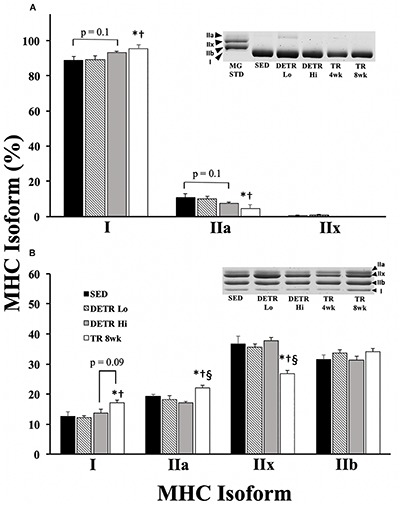
Percent MHC isoform expression in SED, detrained (DETR), and trained (TR) soleus **(A)** and plantaris **(B)** muscles of adult female rats. DETR consisted of 4 weeks of voluntary running followed by 4 weeks of SED conditions and were categorized as high- (DETR Hi) or low- (DETR Lo) total running distances. Exercise-trained rats ran for 8 weeks (TR 8wk). The percentage of each isoform relative to the total MHC content was determined within each muscle; total MHC isolate from rat gastrocnemius muscle (MG STD) was used as a control to observe the migratory patterns of the samples. Representative gels show MHC isoform migration for soleus and plantaris muscles for all groups, including samples from TR 4wk rats (group results presented in Table 2). Values are means ± SEM. Significant differences from SED (*), DETR Lo (†), and DETR Hi (§) are indicated; significance was set a *p* < 0.05.

In the soleus, the predominant MHC isoforms were type I and IIa among all groups (Figure 2A). Soleus muscles from SED and DETR Lo rats were comprised of similar MHC isoforms and contained 88.7 and 89.3% type I, 10.9 and 10% type IIa, and trace levels (<1%) of type IIx, respectively. Compared to SED and DETR Lo rats, the soleus of TR 8wk rats were composed of significantly greater type I (95.3%) and lower type IIa MHC (4.7%). DETR Hi rat soleus muscle trended (*p* = 0.1) toward greater type I (93.3%) and lower type IIa (7.1%) than SED rats. No type IIx MHC was detected in the soleus of DETR Hi or TR 8wk rats.

All four adult MHC isoforms were present in the plantaris of each group (Figure 2B). The plantaris of TR 8wk rats had significantly greater type I (17.1%) and type IIa (22%) MHC content than either SED (12.7% type I and 19.2% type IIa MHC, respectively) and DETR Lo (12.2% type I and 18.3% type IIa MHC, respectively) rats (*p* < 0.05). DETR Hi plantaris muscles had less type IIa MHC (17.2%) than TR 8wk muscles (*p* < 0.05). TR 8wk rat plantaris muscles trended (*p* = 0.1) toward a greater type I MHC content than detected in DETR Hi rats (13.8%). Conversely, the TR 8wk plantaris contained significantly less type IIx MHC (26.8%) than SED (37.7%), DETR Lo (35.7%), and DETR Hi (37.7%) rats (*p* < 0.05).

#### Capillarity

**FIGURE 3:**
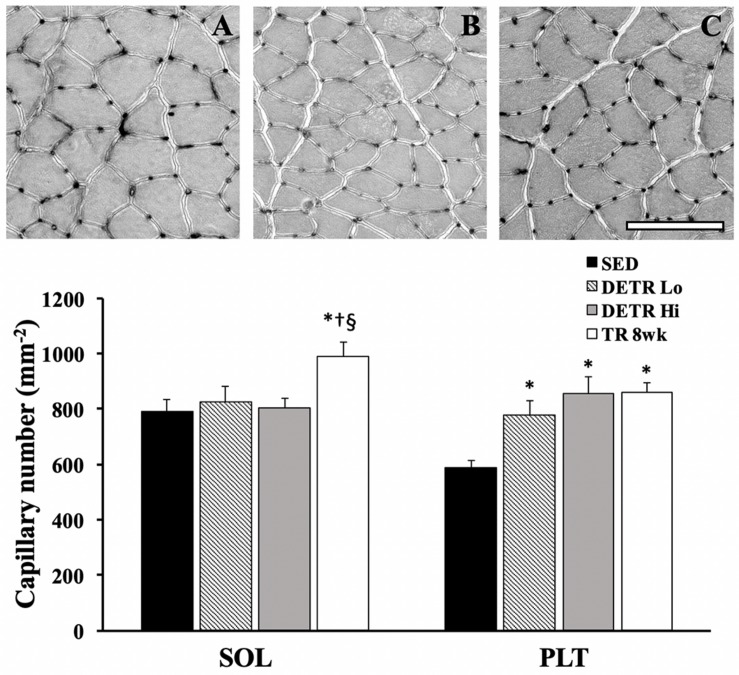
Total capillary number (per mm^2^) in SED, detrained (DETR), and trained (TR) soleus and plantaris muscles of adult female rats. DETR consisted of 4 weeks of voluntary running followed by 4 weeks of SED conditions and were categorized as high- (DETR Hi) or low- (DETR Lo) total running distances. Exercise-trained rats ran for 8 weeks (TR 8wk). Representative lead-nitrate-based histochemistry from SED **(A)**, DETR Hi **(B)** and TR 8wk **(C)** plantaris muscle cross sections are shown; scale bar = 100 μm. Values are means ± SEM. Significant differences from SED (*), DETR Lo (†), and DETR Hi (§) are indicated; significance was set a *p* < 0.05.

Capillary density in soleus and plantaris muscles was expressed as the total number of capillaries per mm^2^ of tissue area (Figure 3). A significantly greater number of capillaries per mm^2^ were present in TR 8wk soleus compared to all other groups (*p* < 0.05). By contrast, the number of capillaries in the plantaris increased significantly in DETR Lo, DETR Hi, and TR 8wk rats above SED levels (*p* < 0.05).

#### Citrate Synthase

**FIGURE 4:**
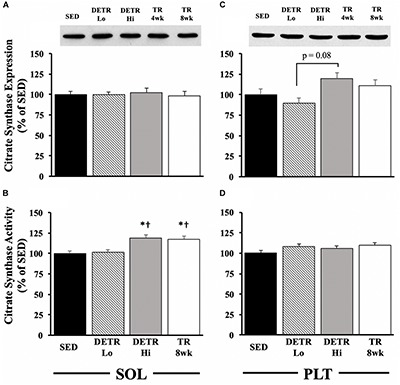
Citrate synthase expression **(A,C)** and activity **(B,D)** in SED, detrained (DETR), and trained (TR) soleus and plantaris muscles of adult female rats. DETR consisted of 4 weeks of voluntary running followed by 4 weeks of SED conditions and were categorized as high- (DETR Hi) or low- (DETR Lo) total running distances. Exercise-trained rats ran for 8 weeks (TR 8wk). Representative blots of citrate synthase protein expression are shown (top) for soleus and plantaris muscles for all groups **(A,C)**, including samples from TR 4wk rats (group results presented in Table 2). Enzyme activity was determined as (μmole/g/min), normalized to protein content (nmoles/mg of protein/min), and then expressed relative to SED values. Values are means ± SEM. Significant differences from SED (*) and DETR Lo (†) are indicated; significance was set a *p* < 0.05.

In the soleus, no differences among groups occurred for citrate synthase protein expression (Figure 4A). In the plantaris, citrate synthase protein expression trended higher in DETR Hi compared to DETR Lo rats (*p* = 0.08; Figure 4C). A higher citrate synthase activity in the soleus was detected in DETR Hi and TR 8wk than either SED or DETR Lo rats (*p* < 0.05; Figure 4B). In plantaris muscles, no differences in enzyme activity were detected among groups (Figure 4D).

#### OXPHOS Proteins

**FIGURE 5:**
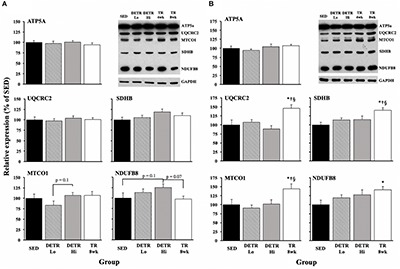
Mitochondrial protein expression in SED, detrained (DETR), and trained (TR) soleus **(A)** and plantaris **(B)** muscles of adult female rats. DETR consisted of 4 weeks of voluntary running followed by 4 weeks of SED conditions and were categorized as high- (DETR Hi) or low- (DETR Lo) total running distances. Exercise-trained rats ran for 8 weeks (TR 8wk). Representative blots for OXPHOS and GAPDH proteins are shown (upper right) for soleus and plantaris muscles for all groups, including samples from TR 4wk rats (group results presented in Table 2). Values are means ± SEM. Significant differences from SED (*), DETR Lo (†), and DETR Hi (§) are indicated; significance was set a *p* < 0.05.

In the soleus, no significant differences among groups were detected (Figure 5A), although DETR Hi rats trended higher in Complex IV - MTCO1 expression than DETR Lo rats (*p* = 0.1) and higher in Complex I – NDUFB8 expression than in SED (*p* = 0.1) or TR 8wk (*p* = 0.07) rats. In the plantaris muscles, TR 8wk rats had significantly greater UQCRC2, MTCO1, and SDHB protein expression than all other groups (*p* < 0.05; Figure 5B). For NDUFB8 protein expression, a difference was only detected between SED and TR 8wk groups (*p* < 0.05; Figure 5B).

### Experiment #2

Comparisons between TR 4wk and TR 8wk groups are shown in Table 2. In the soleus, TR 8wk rats exhibited a greater capillary count than TR 4wk rats (*p* < 0.05). Conversely, a higher expression of the OXPHOS protein Complex I – NDUF8 was detected in TR 4wk than in TR 8wk rats (*p* < 0.05). In the plantaris, type IIx MHC content was greater in TR 8wk (26.8%) than TR 4wk (23.4%) rats (*p* < 0.05). A higher citrate synthase activity was detected in TR 4wk than TR 8wk plantaris muscles (*p* < 0.05). Finally, plantaris muscles from TR 8wk rats had a significantly higher expression of OXPHOS proteins Complex III – UQCRC2 and Complex II – SDHB than TR 4wk rats (*p* < 0.05).

**TABLE 2:** Mean (±SE) total running distance, body and muscle masses, myosin heavy chain (MHC) isoforms, total capillary counts, and mitochondrial proteins in adult female Sprague–Dawley rats after either four (TR 4wk) or 8 weeks (TR 8wk) of voluntary running.

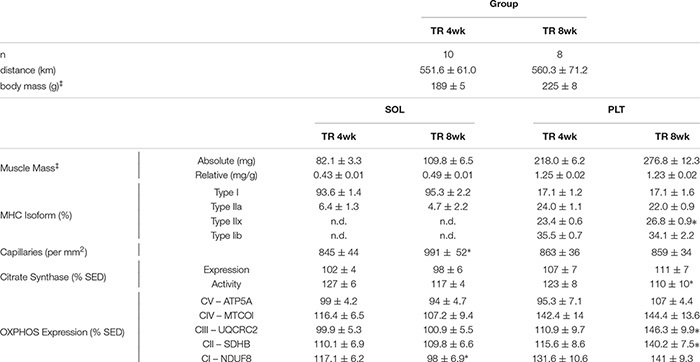

*^‡^No comparisons made due to difference in age between groups. ^∗^Different vs. TR 4wk (*p* < 0.05).*

## Discussion

The goal of this study was to determine the effects of detraining after a period of voluntary wheel running activity within the slower-oxidative soleus and faster-glycolytic plantaris hindlimb muscles in rats. Generally, we show muscle-specific responses to detraining for MHC isoforms, total capillary number, and mitochondrial markers. In agreement with our hypothesis, 4 weeks of detraining clearly reversed the fast-to-slow phenotypic shifts within the plantaris muscle. Contrary to our hypothesis, gains in total capillary number (per mm^2^) in the plantaris were retained after 4 weeks of detraining irrespective of total running distance; in the soleus, gains in capillarity were maintained only in the 8-week trained rats. Mitochondrial markers for aerobicity showed few changes in both trained and detrained soleus muscles, whereas detraining appeared to reverse gains in the plantaris for these aerobic indicators. Finally, comparisons between 4- or 8-week exercising rats provided insight on muscle-specific changes pertaining to exercise intensity and/or duration.

### Myosin Heavy Chain Isoform Expression

It is well established that the rat soleus is predominantly slow-oxidative (type I MHC) whereas the plantaris is a mixed muscle with a majority of type faster-glycolytic (type IIx and IIb MHC) isoforms; this is consistent with the phenotypic profiles of SED rats in this study (Figure 2). In concert with earlier work (Ishihara et al., 1991; Demirel et al., 1999; Bigard et al., 2000; Seene et al., 2005; Fuller et al., 2006), rats that experienced consistent physical activity in the present study (TR 8wk) increased the proportion of slow and/or more oxidative phenotypes (type I and/or IIa isoforms) in each the soleus and plantaris (Figure 2). In the plantaris, the relative increase in type I and IIa isoforms was accounted for by a marked decrease in type IIx MHC.

Ishihara et al. (1991) found no phenotypic changes in soleus or plantaris muscles of male Wistar rats after 45 days of voluntary running (average distance, 1–3 km/day). Similar to our findings, Fuller et al. (2006) reported an enhanced type I MHC content in soleus and plantaris muscles of male Wistar rats after 8 weeks of voluntary running (average distance, ∼5.4 km/day). By comparison, in the present study, TR 8wk rats averaged ∼8.8 km/day, suggesting that the total running distance, or the amount time per day spent physically active, is an important factor impacting the magnitude of the phenotypic changes in the rat soleus and plantaris muscles. Kariya et al. (2004), however, observed gains in type I and IIa MHC in the plantaris of male Sprague–Dawley rats after 10 weeks of voluntary running even though the rats ran less (3 km/day) than DETR Lo (4.4 km/day) or DETR Hi (9.5 km/day) rats in the present study, suggesting that there may be training (duration) threshold for eliciting and sustaining fast-to-slow MHC shifts within the plantaris. Several groups (Demirel et al., 1999; Seene et al., 2005) have noted that the fast-to-slow MHC shifts in rat soleus, plantaris, and extensor digitorum longus muscles are dose-dependent (e.g., min/day). This notion is further supported, in part, by the observation that type I and IIa MHC content in the soleus and plantaris muscles were comparable in TR 4wk and TR 8wk groups that ran similar distances (Table 2).

Myosin heavy chain profiles from DETR Lo and DETR Hi soleus muscles suggest that the quantity of exercise before detraining has some impact on type I and IIa isoform content based on the statistical trends detected between DETR Hi and SED groups. The soleus is active 11–15 h per day during normal postural and cage activity (Hodgson et al., 2005), which may influence the maintenance of prior training adaptations during the 4-week period of detraining. Conversely, in the plantaris, any slow/oxidative gains made during the exercise training period were eliminated by detraining, suggesting that the trained plantaris quickly reverted back to phenotypic profiles that coincided with a neural recruitment profile that matched SED conditions (Gardiner et al., 1986; Jasmin and Gardiner, 1987). Comparisons between TR 4wk and TR 8wk muscles highlight that phenotypic adaptations can occur quickly to meet the demands of exercise (Table 2). Taken together, our findings show that detraining has a significant impact on phenotypic changes accrued during a training period, which is particularly evident within plantaris and, to a lesser degree, soleus muscles. To our knowledge, this is the first study to report profiles of MHC isoforms in detrained rodent skeletal muscle following an aerobic endurance training paradigm.

### Capillary Density

Changes in capillarity with voluntary physical activity and detraining was muscle specific. As expected, the soleus from TR 8wk rats exhibited an increase in capillary number, but no changes were observed for any other groups (Figure 3 and Table 2), suggesting that 4 weeks of voluntary running was insufficient to induce angiogenesis in an already highly vascularized and oxidative muscle. An increased capillarity also was reported by Malek et al. (2010) in the soleus of treadmill-trained male Sprague–Dawley rats after 10 weeks. These findings contrast with Olenich et al. (2014) who showed that capillary-to-fiber ratio in the mouse soleus was enhanced after only 3 weeks of voluntary running. Both groups (Malek et al., 2010; Olenich et al., 2014) showed that the gains in capillary density returned to SED control levels after 1 week of detraining, which may appear to explain why the capillary numbers were similar in DETR Lo, DETR Hi, and SED soleus muscles (Figure 3). Olfert (2016) attributes capillary regression to an upregulation in anti-angiogenic thrombospondin-1 (TSP-1) induced by inactivity rather than a downregulation in pro-angiogenic factors such as vascular endothelial growth factor (VEGF). The expression levels of such factors in trained and detrained muscles would be interesting to assess in future studies.

In the plantaris, a different pattern emerged for total capillary number as compared to the soleus. Generally, all rats that engaged in physical activity, regardless of the total distance accumulated, increased plantaris capillarity (Figure 3 and Table 2). Physical activity has been shown to increase plantaris muscle capillary density after one (Waters et al., 2004), three (Olenich et al., 2014), or 10 weeks (Malek et al., 2010) of training, which agrees with our findings in TR 4wk and TR 8wk rats (Table 2). In humans, Madsen et al. (1993) showed no changes in the capillary-to-fiber ratio after 4 weeks of detraining in the vastus lateralis of highly trained endurance athletes, suggesting that the retention of capillary number during detraining is based, in part, on initial training status when the period of inactivity began (Coyle et al., 1984). Malek et al. (2010) showed that total capillary count (per mm^2^) in detrained plantaris actually increased relative to trained rats (Malek et al., 2010), which also was observed by Prior et al. (2015) in 2-week detrained vastus lateralis of elderly adults.

Collectively, it appears as if the plantaris responds more robustly to alter its capillarity to exercise and is more resistant to reversing the increases in capillary number with detraining than does the soleus (Klausen et al., 1981; Madsen et al., 1993; Waters et al., 2004; Malek et al., 2010; Olenich et al., 2013, 2014, Prior et al., 2015). Our findings coincide with the muscle-specific changes observed in whole-muscle blood flow rates in trained and untrained rats at rest and varying speeds of treadmill running (Laughlin and Armstrong, 1982; Armstrong and Laughlin, 1983, 1984, 1985; Laughlin and Ripperger, 1987). Furthermore, our observations imply that the existing capillary density in an untrained soleus is sufficient to perfuse the muscle during voluntary aerobic training. It is likely that triggering an angiogenic response in the soleus requires some threshold of training volume or duration: the differences in capillarity between TR 4wk and TR 8wk groups suggest that, on a whole-muscle level, this response is duration- rather than intensity-dependent (Table 2). By contrast, the relatively faster plantaris has an insufficient baseline capillary density to meet the demands of the voluntary aerobic activity; hence, the angiogenic response is more pronounced in the plantaris than in the soleus. These adaptations in the plantaris are reflected in a greater relative whole-muscle blood flow response after training than the soleus (Armstrong and Laughlin, 1984). Finally, new capillary growth in the plantaris was sustained with detraining despite the return of MHC phenotype to SED level, suggesting that these muscular characteristics are not temporally synced during (Waters et al., 2004) or after aerobic training.

### Mitochondrial Markers of Aerobicity

Physical activity enhances mitochondrial biogenesis (Holloszy, 1967; Wright et al., 2007; Yan et al., 2011; Booth et al., 2015; Granata et al., 2016; Yokokawa et al., 2018), which is, presumably, reflected by the expression and activity of known metabolic markers associate with the Kreb’s Cycle and/or the electron transport chain. An increase in citrate synthase activity, for example, following an exercise training protocol is well established, and this change appears universal within trained slow and fast muscle phenotypes (Holloszy, 1967; Siu et al., 2003; Fuller et al., 2006; Granata et al., 2016). In the present study, we found this observation consistent in trained (DETR Hi and TR 8wk) soleus muscles, but not in plantaris muscle from the same groups. Siu et al. (2003) observed that citrate synthase activity is highest in 8-week trained rat soleus muscles within 1 h after the last exercise bout and showed a significant decline in activity within 48 h post-training. Similarly, Neufer et al. (1992) reported a return in rat vastus lateralis citrate synthase activity to SED control levels after 6 weeks of forced treadmill running followed by 1 week of detraining. Madsen et al. (1993) found no differences in enzyme activity in 4-week-detrained vastus lateralis muscle samples of highly trained athletes. We found higher citrate synthase activity in TR 4wk than in TR 8wk plantaris muscles (Table 2), suggesting that exercise intensity may impact the response of enzymatic activity within this muscle. We did not observe comparable patterns between citrate synthase protein expression and activity; Siu et al. (2003) reported a similar temporal pattern between mRNA expression and activity in trained rat soleus. Others have reported increases in activity before mRNA and protein expression (Egan et al., 2013), suggesting a discordant relationship between protein accumulation and activity.

Generally, we observed few changes in OXPHOS markers in trained and detrained soleus (Figure 5A), suggesting that the existing mitochondrial density in the untrained higher oxidative soleus is sufficient for the demands required for voluntary physical activity. This observation is consistent with Yokokawa et al. (2018) who showed few changes in mitochondrial OXPHOS proteins in the mouse soleus after one and 8 weeks of voluntary wheel running. However, unlike the soleus, plantaris muscles of TR 8wk rats showed elevated expression of UQCRC2 (Complex IV), MTCO1 (Complex III), SDHB I (Complex II), and NDUFB8 (Complex I) proteins; any gains in these OXPHOS proteins that occurred in the plantaris during training were eliminated with detraining (Figure 5B). In agreement with our results, Yokokawa et al. (2018) reported an upregulation in all plantaris OXPHOS markers after 8 weeks, but not after 1 week, of aerobic training. Comparisons between TR 4wk and TR 8wk plantaris muscles indicate a time-specific sensitivity for the enhanced expression of MTCO1 (Complex III) and SDHB I (Complex II) (Table 2).

Muscle-specific differences in OXPHOS expression have been noted elsewhere (Ikeda et al., 2006), suggesting that the soleus requires a greater training stimulus, possibly associated with exercise achieved in a high-intensity training protocol, than what is required for the plantaris (Hamidie et al., 2015). Indeed, high-intensity training has been shown to increase respiration flux capacity in fibers from human vastus lateralis muscle (Vincent et al., 2015). Taken together, OXPHOS protein expression in trained plantaris appears to parallel the favorable aerobic enhancements observed in MHC phenotypic shifts and capillarity, as well as their reversal with detraining.

### Perspective

High-intensity aerobic interval training (e.g., HIIT) has been shown to be more effective for improving maximal whole-body oxygen consumption (VO_2_max) than moderate exercise intensities (Helgerud et al., 2007). Our findings suggest that the enhancement in VO_2_max result from the recruitment and subsequent adaptations in fast-glycolytic muscle phenotypes because slow-oxidative muscles are likely sufficiently prepared to meet the demands of lower intensity voluntary exercise. Similarly, the rapid loss in aerobic muscular fitness with detraining occurs through the decompensation of fast-glycolytic muscles, although capillary increases appear to be retained when reduced activity follows an initial training period. Expanding understanding of muscle-specific responses to training and detraining may facilitate muscle-specific rehabilitation strategies following injury or within aging populations aimed at enhancing fitness, functional capacities, and fatigue resistance to improve overall quality of life.

## Data Availability Statement

The raw data supporting the conclusions of this manuscript will be made available by the authors, without undue reservation, to any qualified researcher.

## Ethics Statement

The animal study was reviewed and approved by the Institutional Animal Care and Use Committee, University of Puget Sound, Tacoma, Washington.

## Author Contributions

J-PH performed the theory and design, data collection, and analysis and interpretation. EB and HD performed the data collection and analysis. GM performed the design, analysis, and interpretation. All authors contributed to either the drafting or revising the manuscript at different stages and all have approved the final version. All authors agreed to be accountable for all aspects of the work in ensuring that questions related to the accuracy or integrity of any part of the work are appropriately investigated and resolved.

## Conflict of Interest

The authors declare that the research was conducted in the absence of any commercial or financial relationships that could be construed as a potential conflict of interest.
